# Berberine improves lipid levels, vascular function, and gut microbial-dependent metabolites in stable coronary artery disease patients

**DOI:** 10.3389/fnut.2026.1945710

**Published:** 2026-10-20

**Authors:** Honghong Liu, Siqin Feng, Muyun Tang, Jiejun Sun, Ran Tian, Shuyang Zhang

**Affiliations:** 1 Department of Cardiology, Peking Union Medical College & Chinese Academy of Medical Sciences, Beijing, China; 2 Department of Internal Medicine, Peking Union Medical College & Chinese Academy of Medical Sciences, Beijing, China; 3 State Key Laboratory of Complex Severe and Rare Diseases, Peking Union Medical College Hospital, Peking Union Medical College & Chinese Academy of Medical Sciences, Beijing, China

**Keywords:** aromatic amino acid, berberine, coronary artery disease, gut microbiota, lipids metabolism, short-chain fatty acids, vascular endothelial function

## Abstract

Berberine (BBR) has been demonstrated to exert cardiovascular protective effects by regulating lipids and ameliorating inflammation. Recent studies reported the interaction between BBR and gut microbiota in various metabolic diseases due to its low bioavailability. However, few studies examine the effect of BBR on the gut microbiota in coronary artery disease (CAD) individuals. We hence conducted a parallel-controlled, regularly visited clinical trial with 23 stable CAD patients who recently underwent percutaneous coronary intervention and completed a 12-week BBR ± standard therapy treatment. The BBR intervention was associated with a directional improvement in endothelial function, enhanced levels of low-density lipoprotein cholesterol (LDL-C), and a reduction in circulating inflammatory mediators (IL-1α, IL-17, MCP-1, MIP-1β, and VEGF). Using metagenomics and metabolomic studies, we identified that bacterial signatures varied significantly with gradually increasing doses of BBR. These results showed that a specific group of short-chain fatty acid (SCFA)-producing bacteria, mainly *Ruminococcus* sp., *Roseburia* sp., and *Anaerostipes hadrus*, were elevated following the BBR intervention, which was also negatively correlated with inflammatory factors. Notably, regardless of the background standard therapy, BBR reshaped gut-microbiota–encoded aromatic amino acid catabolism: it selectively enriched the microbial L-dopa, 3-hydroxyphenylacetic acid, and phenylpropionic acid branches, which were anchored by increased abundances of dopamine β-monooxygenase, *porA,* and *acdA* genes. Overall, we investigated the role of BBR in regulating microbial metabolic alterations and vascular function. Targeted restoration of the gut microbiota may present a novel ecological approach for managing cardiovascular disease.

## Introduction

Coronary artery disease (CAD), a major burden of cardiovascular diseases, is a chronic inflammatory pathophysiological process influenced by a combination of genetics, lifestyle, and other factors (1). Despite crucial advances in the field of epidemiology and translational research that have enhance our understanding of the pathophysiology of CAD, it continues to be a principal cause of death worldwide (2). An anti-platelet aggregation strategy to treat CAD has potential side effects. Therefore, more suitable and effective drugs should be explored for precise therapy. Recently, the key role of gut microbiota in regulating host metabolism and the associations between gut microbial dysbiosis and the development of atherosclerosis have been extensively explored. Several bacterial metabolic pathways that regulate the production or transport of trimethylamine N-oxide (TMAO) (3), bile acids (4), and short-chain fatty acids (SCFAs) (5) have been implicated in mediating the bacterial regulation of host atherosclerosis. Many butyrate-producing gut bacteria, such as *Faecalibacterium* and *Roseburia* spp., were depleted in patients with CAD (6). Patients with acute coronary syndrome (ACS) exhibited significantly lower plasma levels of butyric acid and propionic acids, and these reductions persisted at the 6-month follow-up (7). Metagenomic and 16S rRNA sequencing studies have consistently demonstrated that CAD patients exhibit reduced α-diversity (8, 9). Our previous study showed that *Bacteroides* spp. were significantly enriched across CAD stages, consistent with their capacity to produce LPS, while *Alistipes* sp., increased specifically in the 3-VD subgroup (10). Therefore, hunting for microbial-targeted remedies for CAD has gained increasing attention.

Berberine (BBR), a natural plant alkaloid extracted from *Berberis aristata* and *Coptis chinensis* (Huanglian), is an ancient anti-diarrheal medication and has been reported to be an effective remedy for metabolic disorders by regulating lipid and glucose metabolism (11). However, the specific *in vivo* target of BBR has barely been clarified, as its poor oral bioavailability suggests a potential effect on the gut microbiome. Preclinical studies demonstrate that BBR suppresses TMAO-producing bacteria (*Clostridiales*), and promotes SCFA producers such as *Roseburia* and *Faecalibacterium prausnitzii* in cardiometabolic diseases, including diabetes and hyperlipidemia (12). Research in animals has established that BBR protects the myocardium through antioxidant and lipid-lowering mechanisms (13). Endothelial function plays a central role in the pathophysiology of stable CAD after percutaneous coronary intervention (PCI); consequently, restoring endothelial function—rather than merely achieving anatomical luminal patency—is increasingly recognized as a critical therapeutic goal (14). However, how berberine affects endothelial function and metabolic phenotypes in CAD patients after PCI and how the human gut microbiome responds to BBR treatment have not yet been investigated.

Thus, the current study was designed to determine the vascular function effects and gut microbiota alterations of berberine administration in stable CAD patients after percutaneous coronary intervention (PCI), and their impact on lipid metabolism, inflammatory status, and microbe-related metabolic pathways.

## Methods

### Study design

This is a single-center, parallel-controlled study that includes a run-in period of 2 weeks and a treatment period of 12 weeks, conducted between 2020 and 2021. This study was approved by the Medical Ethics Committee of Peking Union Medical College Hospital (JS-1500). This trial was registered at ClinicalTrials.gov (Trial No. NCT04434365).

### Study population

We screened stable coronary artery disease patients (15) who received coronary angiography and percutaneous coronary intervention (8–40 weeks) at Peking Union Medical College Hospital. Subjects provided written informed consent for participation in the study. Participants were instructed to maintain their habitual diet/exercise and avoid probiotics/antibiotics. The exclusion criteria are as follows: 1. Planned coronary revascularization, including PCI and coronary artery bypass graft during the study period; 2. Sustained uncontrolled hypertension (systolic blood pressure> 180 mmHg); 3. History of medication dose adjustments for vasodilators and statins within 4 weeks; 4. Use of berberine within 4 weeks before enrollment; 5. History of intolerance to berberine; 6. Significant impairment of liver function, renal function, mental health disorders, severe infections, severe anemia, or neutropenia; 7. Heart failure or LVEF <50%; 8. Sustained uncontrolled arrhythmias; 9. Pregnancy; 10. Presence of a malignant tumor or a life expectancy of less than half a year; 11. Inability to complete follow-up. 12. Consumption of fermented dairy products and probiotics for at least 7 days before the screening. 13. Use of antibiotics or other interventions that could affect the gastrointestinal tract within 2 months before the screening and during the whole study period.

### Intervention plan

After inclusion, 23 screened subjects were screened and underwent baseline data acquisition and guidance (Visit 1). Participants were allocated a 1:1 ratio to either BBR + ST or ST using a computer-generated random sequence (block size 4). To ensure allocation concealment, sealed opaque envelopes were used, which were handled by an independent nurse. Participants received either a berberine intervention (Northeast Pharmaceutical Group Shenyang First Pharmaceutical Co., Ltd., Lot No.: 130917) ± on the basis of CAD standard therapy (Detailed in Table 1). To ensure the safety of the subjects and improve their tolerance, this study started with a small dose and gradually increased the dose as follows: Stage 1: weeks 1–4, 100 mg three times daily (tid); Stage 2: weeks 5–8, 200 mg tid; Stage 3: weeks 9–12, 300 mg tid. During the study, all administered medications and any side effects were recorded in the appropriate sections of the Case Report Form (CRF). Adherence was monitored via pill counts at Visits 2, 3, and 4. Subjects with a pill count indicating <80% adherence were flagged as protocol deviations; however, no participants dropped below this threshold. The visit plan, including the follow-up timeline and clinical assessment details, is summarized in Figure 1a and Table 1.

**Table 1:** Baseline demographics characteristics of participants.

ID	Gender	Age	BMI	Smoke	No. of SV	DM	HTN	HF	PAD	Anti-platelet	Statins	*β* − *B*	ACEI/ARB	OAD
B001	M	61	26.6	Y	1	Y	Y	N	Y	A + T	Y	Y	N	M
B002	M	57	30.1	Y	2	N	Y	N	Y	A + T	Y	Y	Y	N
B003	F	63	23.3	N	2	Y	Y	N	Y	C	Y	Y	Y	N
B004	M	52	29.7	Y	2	Y	Y	N	N	A + T	Y	Y	Y	M
B005	M	65	22.7	Y	3	N	N	Y	Y	C	Y	Y	Y	N
B006	M	41	28.3	N	3	Y	N	N	N	A	Y	Y	N	M
B007	F	42	26.0	Y	1	N	Y	N	N	A	Y	N	N	N
B008	M	56	27.5	Y	3	N	Y	N	Y	A	Y	Y	N	N
B009	F	62	25.8	Y	3	N	Y	N	N	A	Y	Y	Y	N
B010	M	40	26.7	Y	2	Y	Y	N	N	A	N	Y	N	N
B011	F	64	22.4	Y	2	N	Y	N	N	A	Y	Y	Y	N
B012	M	64	23.5	N	3	Y	Y	N	N	A	Y	N	Y	A
C001	F	79	21.5	Y	2	N	Y	N	N	A + C	Y	Y	N	N
C002	F	78	25.9	N	2	Y	Y	Y	Y	A + C	Y	Y	Y	N
C003	M	65	24.5	N	1	N	Y	N	N	A + C	Y	N	Y	N
C004	F	65	31.6	N	3	N	Y	N	N	A + C	Y	Y	N	N
C005	F	78	28.4	N	3	N	Y	N	N	A + C	Y	Y	Y	N
C006	F	70	24.3	N	3	Y	Y	N	N	A + C	Y	Y	Y	M
C007	M	70	24.6	Y	3	N	N	N	N	C	Y	N	N	N
C008	M	57	23.5	N	2	N	N	N	Y	A	Y	Y	N	M
C009	F	62	27.3	Y	3	Y	Y	N	N	A	Y	Y	Y	N
C010	F	60	24.6	N	1	N	N	N	N	A	N	Y	Y	N
C011	M	59	31.7	Y	2	N	Y	N	N	A	Y	Y	N	N

Y, yes; N, no; Gender: M, male; F, female; BMI, Body mass index; No. of SV, Number of stenosed vessels in coronary artery disease; DM, Diabetes mellitus; HTN, Hypertension; HF, Heart failure; PAD, Peripheral artery disease; Antiplatelet drugs: A, Aspirin; C, Clopidogrel; T, Ticagrelor; ACEI/ARB, Angiotensin-converting enzyme inhibitors/Angiotensin receptor blockers; OAD, Oral antidiabetic drugs; M, Metformin; A, Acarbose.

**Figure 1:**
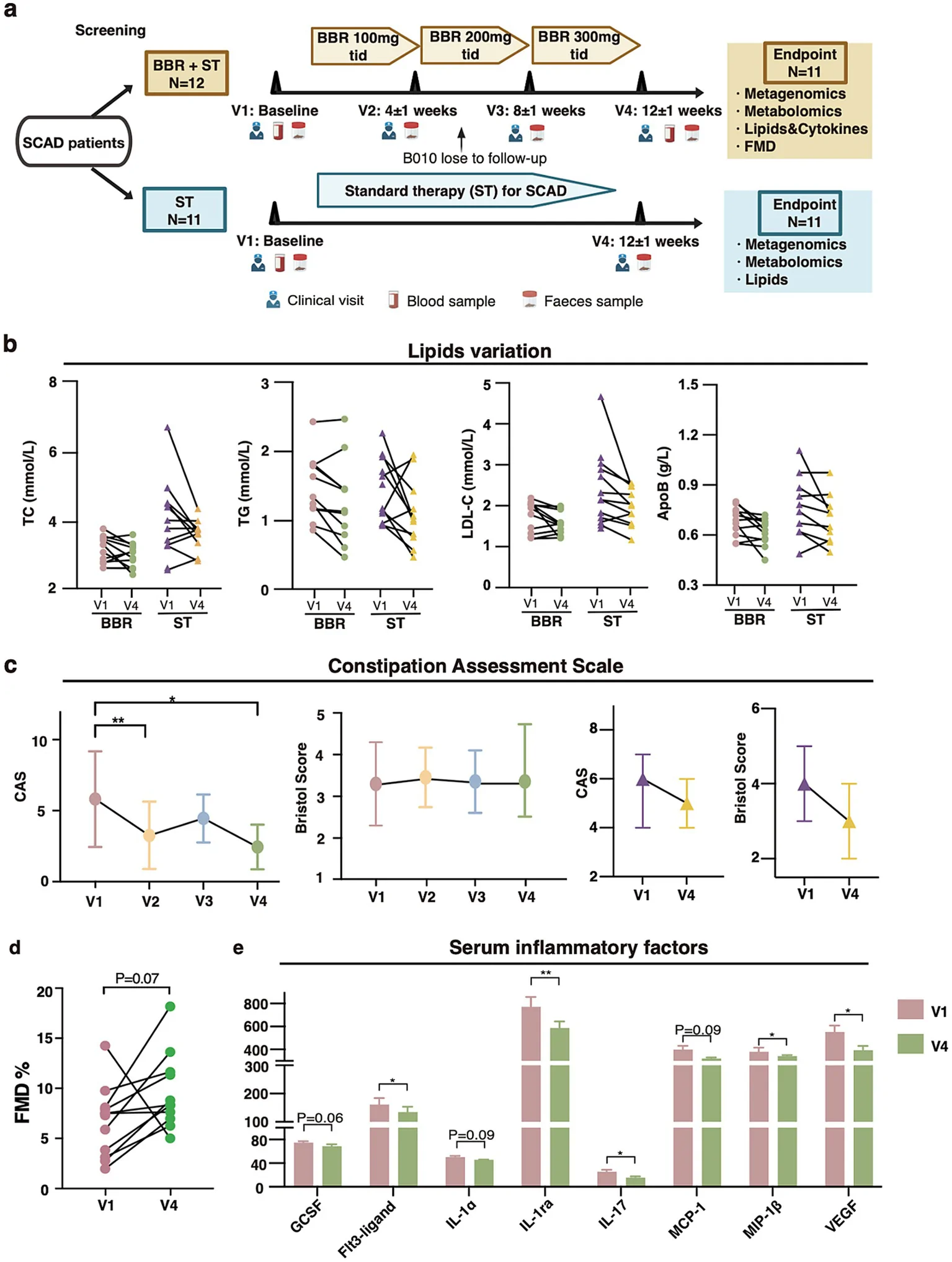
Study design and clinical outcomes after BBR intervention. **(a)** Flow diagram of participant enrolment in the trial. **(b)** Serum lipid variation. **(c)** Self-reported constipation assessment scale and Bristol score at V1-V4 time points. **(d)** Percentage of flow-mediated dilation in participants at V1 (baseline) and V4 (BBR intervention after 12 weeks) time points. **(e)** Representative pro-atherosclerosis inflammatory cytokines in V1 and V4 timepoint in BBR + ST group. For b and d, the dots represent the absolute values for each subject; for e, data are presented as the means ± SEM. These data were analyzed by a paired Student’s *t*-test. For c, data are presented as medians and interquartile ranges (IQRs) and were analyzed by Mann–Whitney *U*-test. *p < 0.05, **p < 0.01.

### Metadata measurements

Endothelial function was reported as percent flow-mediated dilation (FMD%), as previously described (16); all image analyses were performed by trained technicians who were blinded to the subject’s intervention status. Our technique for measuring brachial FMD% has been demonstrated to be highly reproducible. Blood pressure was measured using a standard mercury sphygmomanometer by specially trained nurses when the patient was in a seated position after ≥5 min of rest. Participants’ body mass index (BMI) was calculated as weight (kg) divided by squared height (m^2^). In face-to-face questionnaire interviews, we collected host metadata, including data regarding gastrointestinal distress, defecation, and Bristol stool scores. Peripheral fasting blood was drawn in the morning the day after admission; blood samples were centrifuged at 3,000 rpm for 5 min after standing at room temperature for at least 30 min, and the supernatant was purified. Then, the laboratory data were measured immediately. Inflammatory cytokines were measured by Luminex® Performance Assay human XL cytokine fixed panel (Catalog Number LKTM014), following manufacturer’s instructions.

### Metagenomics study

Participants were given a stool sampler along with detailed, illustrated instructions for sample collection. Stool samples freshly collected from each participant were immediately stored on dry ice and frozen at − 80 °C. DNA was extracted from fecal samples as previously described (17), and was sequenced using the Illumina HiSeq X Ten platform (Beijing, China). Cluster generation, template hybridization, isothermal amplification, linearization, and blocking denaturing and hybridization of the sequencing primers were performed according to the workflow specified by the service provider. Libraries were constructed with an insert size of approximately 500 bp, followed by high-throughput sequencing to obtain paired-end reads with 150 bp in the forward and reverse directions. As shown in Supplementary Table 2, each sample yielded 8.10–10.90 Gb of clean bases, with a clean rate of >92.57%; low-quality and ambiguous bases were filtered out with SOAPnuke v1.5.6. To remove human host DNA contamination, reads were aligned to the human genome reference (hg19) by SOAPaligner v2.22, and the mapping reads were discarded. We co-assembled high-quality paired-end reads using MegaHIT v.1.1.3 into contigs. The co-assembled contigs were binned using the default settings in the “Binning module” implemented in MetaWRAP v1.2. Taxonomy was performed by matching non-redundant bins with the NCBI genome database available as of August 2019 (http://www.ncbi.nlm.nih.gov/, including Bacteria, Archaea, fungi, and viruses). Genes were predicted using MetaGeneMark v.3.26, and a non-redundant gene catalog was constructed with CD-HIT v4.6.4.

To estimate the genera richness of the sample, we calculated the within-sample α-diversity using the Shannon index based on species profiles. Pielou’s evenness (J) is defined as *J* = *H*′/ln(*S*), where H′ is the Shannon diversity index and S is the observed number of species. Values range from 0 to 1, with higher values indicating a more equitable distribution of reads among taxa. For intra-group comparisons, we performed a Wilcoxon matched-pair signed-rank test; for inter-group comparisons, we used the Mann–Whitney *U*-test. Principal coordinates analysis (PCoA) was performed on the species level using Euclidean distance (Centered Log Ratio transformation). Permutational multivariate analysis of variance (PERMANOVA) was used to test for statistical significance between the subgroups using 9,999 permutations. Metagenomic data were metabolically profiled using HUMAnN, and the reads were aligned to the KEGG database, from which the sequences of eukaryotes were excluded. The alignments were transformed into BAM format with SAMtools v1.19.2 and analyzed with HUMAnN v3.9 to obtain the abundance of pathways. Multiple-testing correction was performed within each predefined analysis family using the Benjamini–Hochberg method. These analyses included host-factor PERMANOVA/Adonis screening, differential-abundance analyses, bacteria–phenotype correlations, and KEGG module-level associations. Unless otherwise specified, FDR-adjusted *q* < 0.05 was considered statistically significant.

### Fecal metabolomics study

A total of 25 mg of the sample was weighed and placed into a 2 mL Eppendorf (EP) tube. Then, 700 μL of the extraction agent (MeOH: H_2_O = 4:1, containing internal standards) was added to the sample, and two 5 mm beads were added to the mixture. The samples were homogenized at 40 Hz for 4 min and were sonicated in an ice-water bath for 5 min. This cycle of homogenization and sonication was repeated three times. After that, the samples were incubated at −40 °C for 1 h. Then, all the samples were centrifuged at 12,000 rpm and 4 °C for 15 min. The supernatant of each sample was transferred to a glass vial for LC–MS/MS/MS analysis. An equal amount of each sample was pooled to create a quality control (QC) sample.

The samples were thawed in an ice-water bath and were vortexed for 30s. A total of 100 μL of liquid samples was mixed with 400 μL methanol containing internal standards, and the mixture was vortexed for 30 s. After sonication in an ice-water bath for 15 min, the samples were incubated at −40 °C for 1 h. Then, the samples were centrifuged at 12,000 rpm and 4 °C for 15 min; 200 μL supernatant of each sample was transferred to a glass vial for LC–MS/MS/MS analysis. An equal amount of each sample as a QC sample was pooled.

Different volumes of each standard stock solution were transferred to a flask to form a mixed working standard solution. A series of calibration standard solutions were then prepared by stepwise dilution of this mixed standard solution (containing isotopically labeled internal standard mixture at identical concentrations to the samples).

The LC separation was carried out using a UPLC System (Agilent 1,290 UPLC), equipped with an ACQUITY UPLC BEH C18 Column (1.7 μm, 2.1 mm * 150 mm). The mobile phase A consisted of 0.1% FA in H_2_O, and the mobile phase B was a mixture of H_2_O and MeOH (5:95) containing 10 mmol/L ammonium acetate. The autosampler temperature was set at 6 °C, and the injection volume was 1 μL. An AB Sciex QTrap 6,500 plus mass spectrometer was used for assay development. The typical ion source parameters were as follows: Ion spray voltage, +5,000 V/−4,500 V; curtain gas, 35 psi; temperature, 450 °C; ion source gas 1, 50 psi; and ion source gas 2, 50 psi.

Raw data files generated by LC–MS/MS were processed using SCIEX Analyst Workstation software (1.7.3); metabolite quantification was analyzed using automated analysis software BIOTREE Bio Bud (v2.0.3) (18).

### Statistical analysis

GraphPad Prism 9.0 (GraphPad Software) and SPSS 25.0 were used for statistical analyses. Continuous variables are presented as mean ± SD (or mean ± SEM where indicated). Baseline characteristics were compared between arms with independent *t*-tests or the Mann–Whitney *U*-test (two-tailed) for non-normally distributed variables and *χ*^2^ tests for categorical variables. Longitudinal changes in bioclinical parameters from V1 to V4 were primarily examined by two-way repeated-measures ANOVA (group × time), with the treatment group (BBR + ST vs. ST) as the between-subject factor and visit (V1, V4) as the within-subject factor; because one participant in the BBR + ST arm had a missing V4 LDL-C measurement, a linear mixed-effects model fitted by restricted maximum likelihood (REML) with subject as a random intercept was prespecified as the primary analysis, and a complete-case repeated-measures ANOVA was performed as a sensitivity analysis. For outcomes that were not normally distributed or where sphericity could not be assumed in *post hoc* sub-analyses, non-parametric within-group (Wilcoxon signed-rank) and between-group (Mann–Whitney *U*) tests were additionally performed. Pairwise intra-group comparisons at single time points were further confirmed by paired Student’s *t*-tests (two-tailed). Correlations involving gut microbiota taxa were assessed by non-parametric Spearman’s rank correlation. Where multiple comparisons were conducted across microbiome features, the Benjamini–Hochberg procedure was conducted to control the false discovery rate (FDR < 0.05). Two-sided *p*-value of < 0.05 was considered statistically significant for all primary analyses.

## Results

### Participants and clinical outcomes after BBR intervention

The study initially comprised 23 stable coronary artery disease (SCAD) patients who successfully underwent PCI (between 8 ~ 40 weeks) from Peking Union Medical College Hospital (PUMCH). After screening, 12 SCAD patients were assigned to the berberine + standard therapy (BBR + ST) group; the remaining 11 participants received standard therapy (ST) for coronary artery disease. We collected demographic data, metabolic and inflammation data, fecal metagenomics and metabolomics at baseline (V1) and after a 12-week follow-up (V4) to investigate the role of BBR in the gut microbiome. By the end of the intervention, one subject (B010) in the BBR + ST group voluntarily withdrew. The subjects in the BBR subgroup received berberine 100 mg p.o. tid for 4 ± 1 weeks (Stage 1); then, it was increased to 200 mg p.o. tid for 4 ± 1 weeks (Stage 2), and finally to, 300 mg p.o. tid for 4 ± 1 weeks (Stage 3) in addition to standard treatment (Figure 1a). Baseline demographics are summarized in Table 1. In the BBR + ST group, 66.7% (8/12) were men, with an average age of 55.6 ± 2.7 years, while in the ST group, 36.6% (4/11) were men, with an average age of 67.5 ± 2.4 years. We used flow-mediated vasodilation (FMD) (16) measurements to assess endothelial function and found that the BBR intervention was associated with a trend toward improved FMD (6.7 ± 0.9 vs. 9.7 ± 1.1, *p* = 0.07) (Figure 1d). Two-way mixed-effects ANOVA (Group × Time) was used to assess lipid changes from V1 to V4. A significant interaction was found for LDL-C (*p* = 0.027), with the BBR + ST group exhibiting a greater LDL-C reduction than ST (1.74 → 1.55 vs. 2.33 → 2.01 mmol/L; between-group difference at V4: *p* = 0.028). TC showed only a marginal interaction (*p* = 0.057), indicating that the LDL-C benefit was relatively specific rather than a universal lipid-lowering artifact (Figure 1b; Table 2). In addition, we discovered that BBR effectively regulated bowel function, as indicated by a reduction in self-reported constipation assessment scale (CAS) (19) scores between V1 and V4 (*p* = 0.01). Meanwhile, the Bristol stool form scale showed no significant differences (Figure 1c). Finally, several classical pro-inflammatory cytokines, such as IL-1α, IL-17, MCP-1, MIP-1β (CCL4), and VEGF, were reduced at V4 compared to V1, showing that BBR has anti-inflammatory effects in metabolic disorders (Figure 1e). In this parallel-controlled study, we demonstrated that, in addition to conventional secondary prevention for CAD, BBR provides additional significant benefits in lipid reduction, inflammation suppression, and improvement in vascular endothelial function.

**Table 2:** Clinical laboratory data after BBR intervention.

Clinical parameters	ST			BBR		
	V1 (*n* = 11)	V4 (*n* = 11)	*p* value	V1 (*n* = 12)	V4 (*n* = 11)	*p* value
ALT, U/L	25.2 ± 4.6	24.3 ± 3.2	0.88	21.6 ± 4.6	23 ± 9	0.40
AST, U/L	25.9 ± 4.8	20.4 ± 1.6	0.34	22.6 ± 3.7	21.4 ± 2.3	0.47
TBil, μmol/L	10.7 ± 1.5	13.8 ± 1.6	0.12	14.4 ± 3.5	13.5 ± 4.7	0.43
ALP, U/L	71.7 ± 4.3	65.1 ± 4.8	0.34	76 ± 4.5	85.4 ± 6.7	0.02
TBA, μmol/L	7.3 ± 0.8	5.4 ± 1.8	0.17	3.1 ± 0.4	2.3 ± 1.4	0.08
Creatine, μmol/L	84.8 ± 6.5	74.4 ± 5.4	0.12	84.5 ± 5.9	75.9 ± 4.1	0.004
FBG, mmol/L	8.8 ± 0.9	5.7 ± 0.3	0.01	6.2 ± 0.5	6.2 ± 0.4	0.34
UA, μmol/L	308 ± 11.9	354.5 ± 25	0.08	346.8 ± 13.8	350 ± 20	0.84
TC, mmol/L	4.1 ± 0.3	3.6 ± 0.1	0.2	3.2 ± 0.5	3 ± 0.4	0.12
TG, mmol/L	1.5 ± 0.1	1.2 ± 0.1	0.13	1.5 ± 0.1	1.3 ± 0.2	0.02
HDL-C, mmol/L	0.9 ± 0.06	0.9 ± 0.08	0.7	0.9 ± 0.04	1.03 ± 0.08	0.26
LDL-C, mmol/L	2.3 ± 0.2	1.9 ± 0.1	0.5	1.7 ± 0.1	1.5 ± 0.08	0.08
ApoB, g/L	0.7 ± 0.04	0.6 ± 0.04	0.25	0.7 ± 0.03	0.6 ± 0.03	0.065
FFA, μmol/L	510.8 ± 60	555.8 ± 36.9	0.75	375.2 ± 36.5	470.8 ± 64.7	0.184
Hs-CRP, mg/L	8.5 ± 1.6	0.7 ± 0.08	0.03	1.2 ± 0.2	0.7 ± 0.2	0.09

Data are shown as the mean ± S.E.M. and analyzed followed by paired *T* test. V1, Visit 1 (Baseline); V4, Visit 4 (12 ± 1 weeks); ALT, Alanine aminotransferase; AST, Aspartate aminotransferase; TBil, Total bilirubin; ALP, Alkaline phosphatase; TBA, Total bile acid; FBG, Fasting blood glucose; UA, Uric acid; TC, Total cholesterol; TG, Triglycerides; LDL-C, Low-density lipoprotein cholesterol; ApoB, Apolipoprotein B; FFA, Free fatty acid; hs-CRP, High-sensitivity C-reactive protein.

### Metagenomic analysis showed a significant impact of BBR on the gut microbiome of SCAD participants

Shotgun metagenomics sequencing was performed on 68 fecal samples (46 in the BBR + ST group and 22 in the ST group), which led to a catalog of 3,094,494 non-redundant microbial genes (Detailed in Methods, Supplementary Table 2). We found that the baseline α-diversity of the BBR + ST group was similar to that of the ST group; the diversity of the gut microbiota was enriched with BBR intervention (Figure 2a). Our data are consistent with the current notion that greater overall diversity implies better health. Based on the Bray–Curtis distance at the species level, the overall structure of the gut microbiota showed significant alteration between comparisons from V1 to V4 in the BBR + ST group. Similarly, marked differences between the BBR + ST and. ST groups were also evident at V4 (*p* = 0.018, PERMANOVA test, Supplementary Table 3; Figure 2b). Compared to the ST group, a total of 43 species changed their relative abundances after 12 weeks of the BBR intervention; 17 were designated as the key BBR-responsive taxa (Wilcoxon matched-pair signed-rank test, *q* < 0.05, Figure 2c; Supplementary Table 4). With the BBR intervention, the abundance of *Megasphaera micronuciformis*, *Megasphaera elsdenii*, *Clostridiales bacterium*, and *Azospirillum* sp. was significantly reduced. We found that *Megasphaera micronuciformis* was significantly negatively correlated with total blood lipids, including TC, lipoprotein(a) (Lpa), and C-reactive protein (CRP) levels (Figure 2d). However, *Ruminococcus* sp., *Roseburia* sp., and *Anaerostipes hadrus* increased with the BBR intervention, showing no variance in the ST group. *Ruminococcus* sp. showed a positive correlation with LDL-C levels in both the BBR + ST and ST groups, and *Roseburia* sp. was positively correlated with FFA. *Roseburia* sp. and *Ruminococcus* sp. were well known for producing short-chain fatty acids (SCFAs) from fermenting polysaccharides or oligosaccharides in the gut (20). Surprisingly, the abundances of *Flavonifractor plautii* and *Eubacterium ventriosum* (famous for production of SCFAs in the human gut) (21) were decreased under standard CAD treatment, but increased following the intervention combined with BBR. *Flavonifractor plautii* belongs to Clostridiales; *F plautii* and its relative metabolite cis-aconitic acid have been shown to protect against elevated arterial stiffness by improving the elastic fiber network and reversing increased pulse wave velocity (22). In conclusion, the BBR intervention led to pronounced alterations in the gut microbiota structure, thereby driving a substantial increase in the abundance of short-chain fatty acid-producing strains.

**Figure 2:**
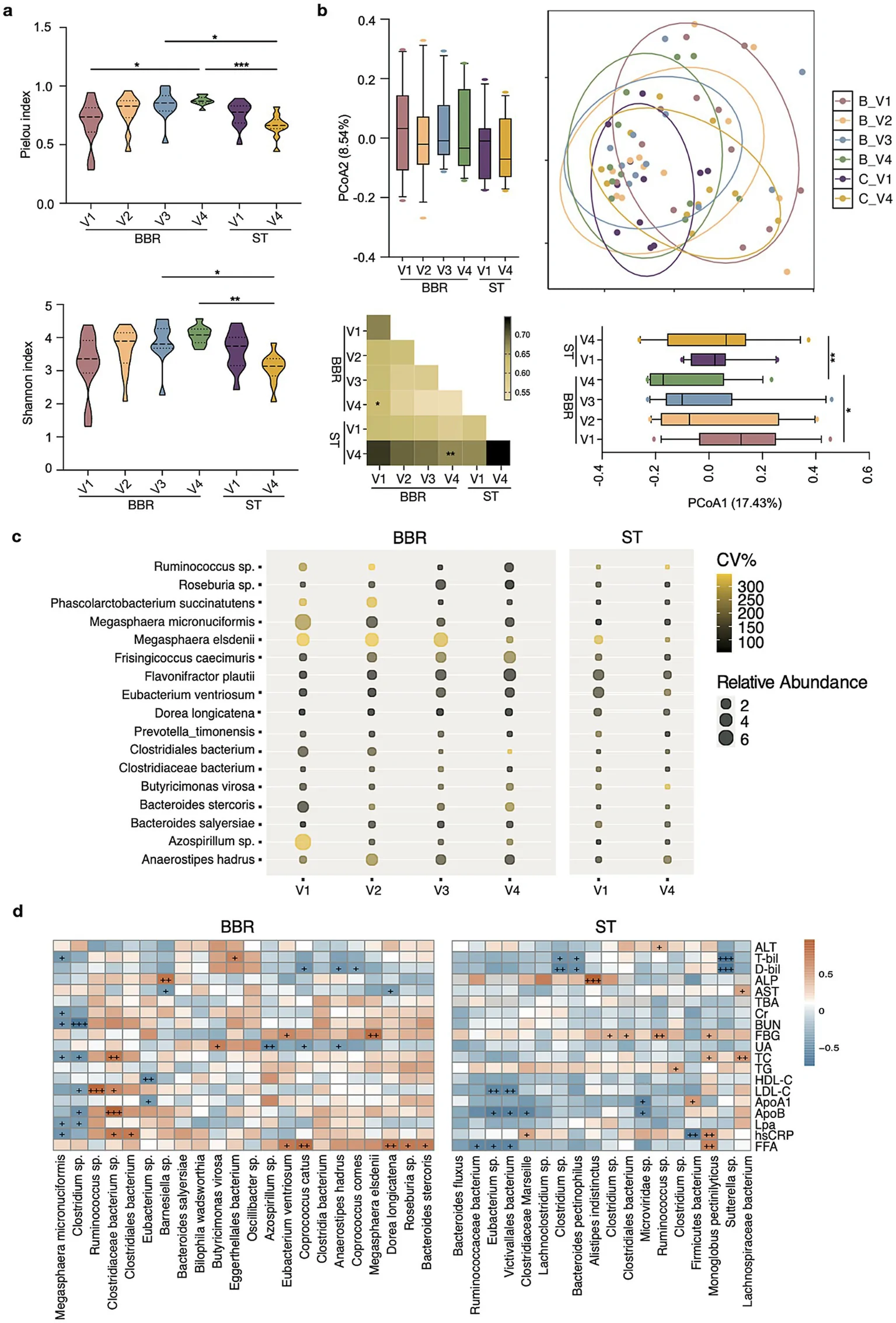
Gut microbial variation after BBR intervention. **(a)** α-diversity analysis at Shannon and Pielou indices was altered by BBR intervention. **(b)** Overall gut microbial structure. Principal coordinates analysis was performed on the basis of the Euclidean distance. Statistical significance was calculated by PERMANOVA, *p < 0.05, **p < 0.01. **(c)** Species significantly changed (Wilcoxon rank sum test, *q* value < 0.05) after BBR intervention. **(d)** Spearman correlation between altered species and clinical indicators. The color represents positive (red) or negative (blue) correlations, and FDRs are denoted as follows: ^+^, FDR < 0.05; ^++^, FDR < 0.01; ^+++^, FDR < 0.001.

### Fecal metabolomics identifies BBR-associated changes in aromatic amino acid and SCFA metabolism

As certain products of fermentation from the gut microbiota could influence host physiology, we explored the fecal metabolomics using LC–MS/MS-MRM-based targeted metabolomics. Based on a principal component analysis (PCA) model, we found that the fecal metabolites were separated (PERMANOVA test, *p* = 0.039, Figure 3a; Supplementary Table 5) between baseline and 12 weeks after standard therapy (ST), while the overall features of metabolites showed no significant difference before and after the BBR intervention. We focused on the main changing pathways after the BBR intervention through KEGG enrichment analysis, including arginine biosynthesis, pyruvate metabolism, and phenylalanine and tyrosine metabolism (Figure 3b). In response to the key altered metabolic pathways, we further compared the differences in metabolites before and after the intervention (Supplementary Table 6). Targeted fecal metabolomics revealed distinct metabolic trajectories following the BBR intervention. Tyrosine and L-tryptophan increased at V4, regardless of BBR exposure, indicating a shared trajectory driven by ST rather than BBR specificity (Figure 3c). In contrast, the BBR intervention induced a significant Group × Time interaction for L-dopa, resulting in significantly higher levels at V4 (3.67 vs. 1.05, *p* = 0.006). L-arginine, 3-hydroxyphenylacetic acid, and phenylpropionic acid followed a concordant BBR-enriched pattern at V4 (L-Arg: 22.6 vs. 9.8, *p* = 0.041). As BBR led to an increase in certain bacteria that produce short-chain fatty acids (SCFAs), we further compared the levels of fecal SCFAs at four different time points in the BBR + ST group (Figure 3d). Acetic acid concentrations showed an increasing trend at V3 and decreased afterward to levels marginally higher than baseline, whereas butyric acid (V1: 11.5, 7.7–18.3; V4: 21.3, 12.6–27.1; *p* = 0.04) and isobutyric acid (V1: 1.1, 0.3–2.5; V4: 1.4,1,1–2.7; *p* = 0.037) concentrations increased significantly at V4. We used the abundance of genes encoding key enzymes to indicate the enrichment of production pathways, specifically *fhs* for acetate and *but* for butyrate formation. The production pathways for acetic acid were gradually enriched in response to the BBR intervention. Normally, four distinct pathways contribute to butyric acid production, and *but* functioned as the terminal gene in the human gut increased significantly after BBR treatment (Figure 3f). Subsequently, we investigated the associations between markedly altered metabolites and biochemical parameters across groups (Figure 3e). It was observed that aromatic amino acids, specifically L-dopa (*rho* = 0.4, *fdr* = 0.02), hydroxyphenyl lactic acid (*rho* = 0.36, *fdr* = 0.03), L-phenylalanine (*rho* = 0.37, *fdr* = 0.046), and imidazole propionic acid (*rho* = 0.46, *fdr* = 0.005), exhibited significant positive correlations with serum lipids (LDL-C and ApoB1). Overall, fecal metabolomics analysis revealed that the BBR intervention enhanced the metabolic activity of aromatic amino acids and short-chain fatty acids (SCFAs).

**Figure 3:**
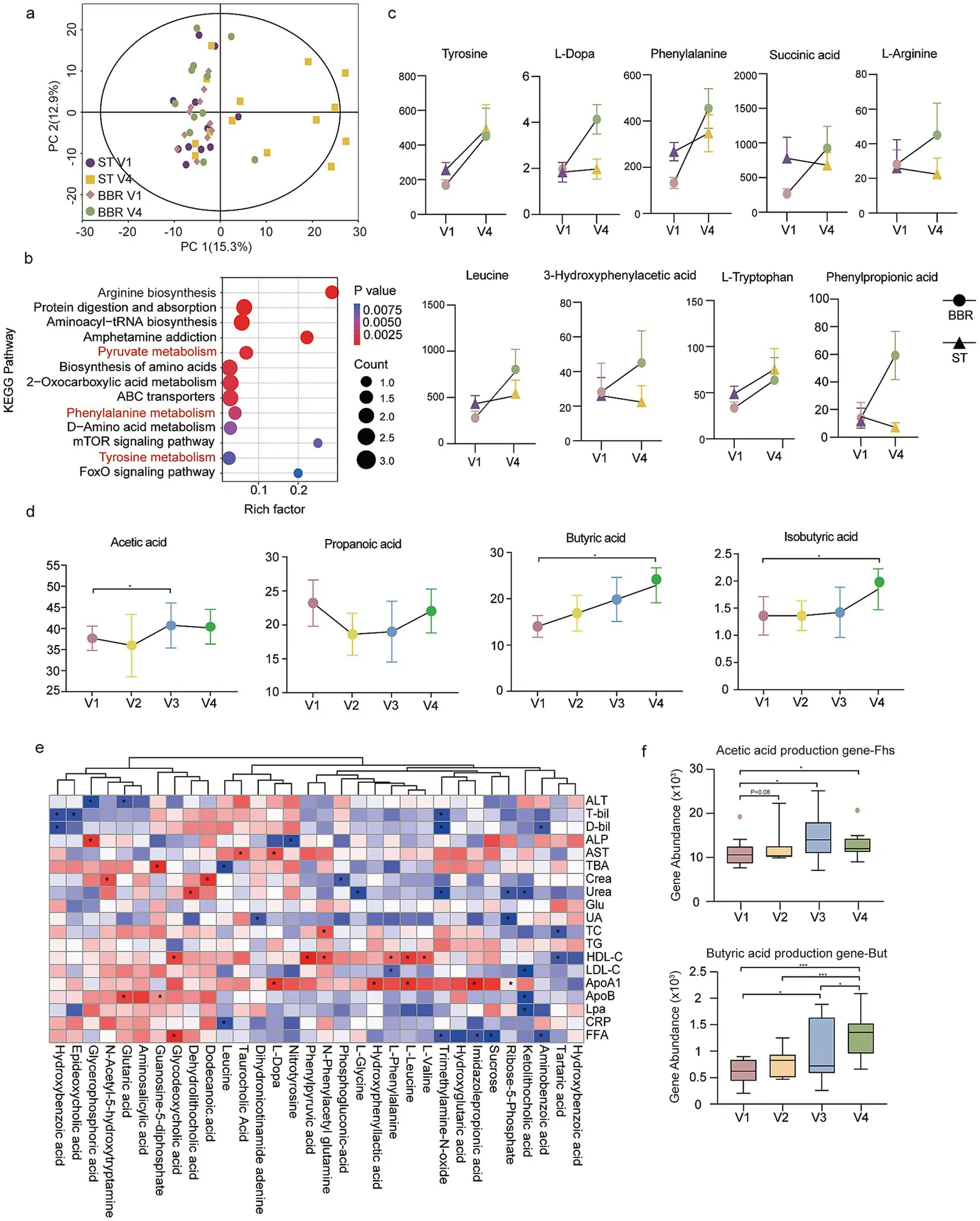
Fecal metabolomics after BBR intervention. **(a)** Principal coordinates analysis of metabolite structures between different groups ± BBR intervention. **(b)** Metabolomic KEGG pathway after BBR intervention. **(c)** Significantly altered fecal metabolites at V1 and V4 time points in BBR ± ST groups. **(d)** Changes in fecal concentrations of SCFAs at 4 time points in the BBR group. **(e)** Spearman correlations between significantly changed species and major coronary artery disease clinical phenotypes at V1 and V4 time points. **(f)** Changes in the abundance of genes that encode the key enzymes in acetic acid production (formate-tetrahydrofolate ligase) and butyric acid production (represented by but). Data are presented as medians and IQRs and were analyzed by Mann–Whitney *U*-test. *p < 0.05; **p < 0.01; ***p < 0.001.

### BBR regulates aromatic amino acid metabolism by remodeling intestinal microbiota

We then conducted a gene-centric analysis of the metagenomic datasets to explore the functional changes in the gut microbiota that might contribute to improved host clinical outcomes under the BBR intervention. The metabolic potential of the gut microbiota was represented by KEGG modules, which are manually curated pathway units consisting of KOs. Seventeen KEGG modules were remarkably altered after the BBR intervention (Figure 4a). We have observed that BBR treatment enhanced multiple aromatic amino acid metabolic pathways, such as those for tyrosine, phenylacetate, phenylalanine, and tryptophan biosynthesis, which also showed a positive correlation with corresponding fecal metabolites. Conversely, the BBR intervention led to a decrease in the abundance of certain metabolic pathways, such as cinnamate degradation, L-histidine catabolism, and leucine catabolism. According to the KEGG metabolic pathways, we mapped out the pathway of aromatic amino acid metabolism. The BBR intervention had little effect on the substrates, phenylalanine and tyrosine; however, the abundance levels of several downstream products, including L-dopa and phenylpropionic acid, were found to be increased (Figure 4b). Pursuing our central hypothesis that aromatic amino acid generation is one of the key mediators of the observed effects of the microbiota after the BBR intervention, we specifically examined the genes involved in the production of these metabolites. We found that dopamine β-monooxygenase gene abundance in the gut microbiota of the BBR + ST group showed a significant correlation with L-dopa (*Rho = 0.6*, *Pval = 0.04*). Likewise, the abundance of the *porA* gene was more strongly correlated with phenylacetic acid (*Rho = 0.7*, *Pval = 0.03*), and the *acdA* gene level also exhibited a stronger correlation with phenylpropionic acid (*Rho = 0.6*, *Pval = 0.04*) under the BBR intervention (Figure 4c). In summary, the shifts in the abundance of genes encoding the aromatic amino acid production pathways were largely consistent with the measured fecal aromatic amino acid content. These results illustrate that berberine is associated with changes in the aromatic amino acid pathway, which is consistent with microbiota remodeling.

**Figure 4:**
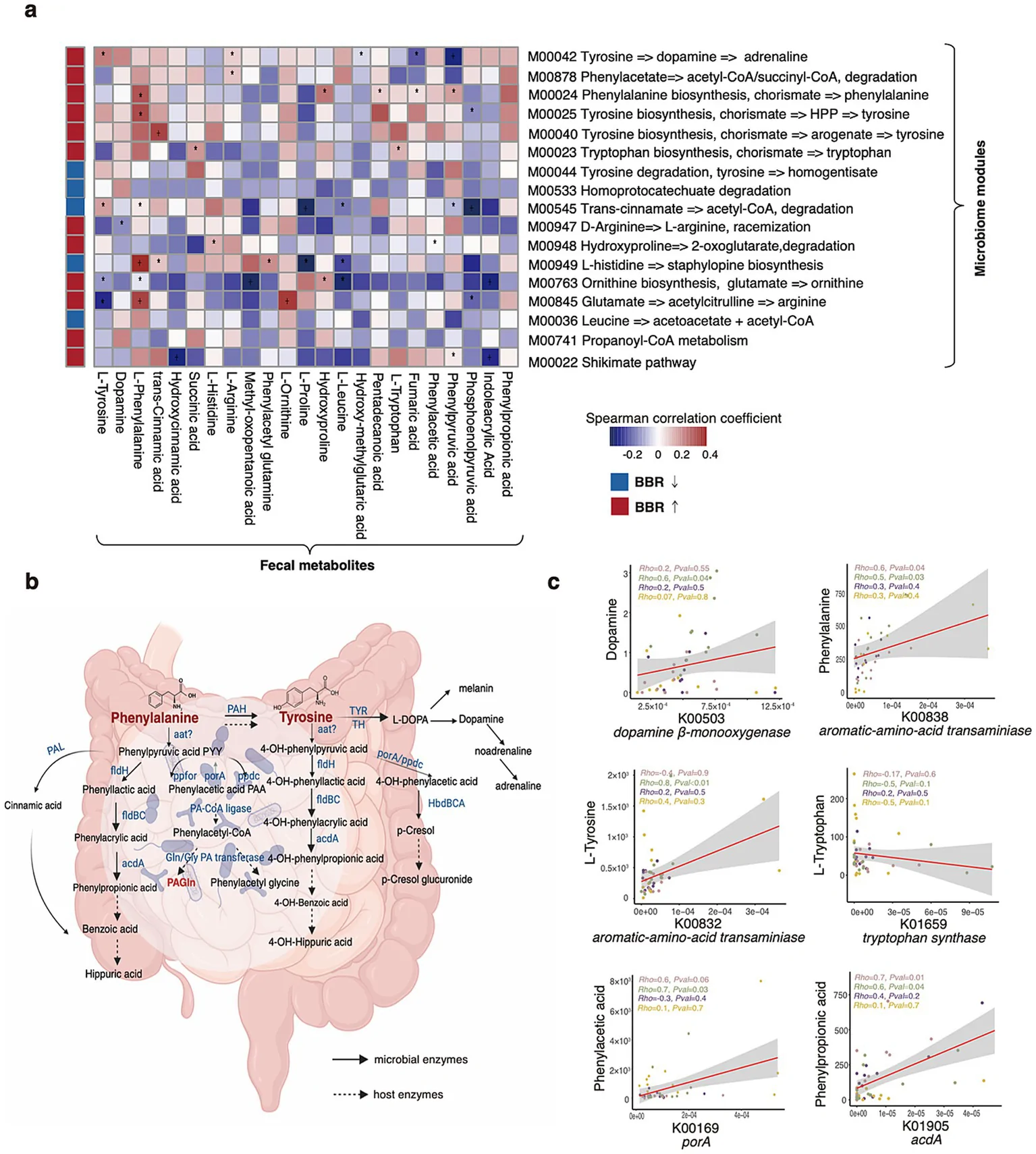
Berberine orchestrates host–microbiota co-metabolism of aromatic amino acids. **(a)** Correlation profile between microbial KEGG modules and fecal metabotypes; coloring represents the Spearman correlation coefficient, where FDRs are denoted: +FDR < 0.05; *FDR < 0.01. The left panel indicates that KEGG modules were either upregulated or downregulated by BBR intervention (Denoted by color). **(b)** Partial gut microbiota involved in the aromatic amino acid pathway related to BBR. **(c)** Spearman correlations between key microbial genes and corresponding fecal metabolites in BBR ± ST groups.

## Discussion

In this parallel-controlled, regularly visited clinical trial conducted among 23 stable coronary artery disease (SCAD) patients who received secondary prevention therapy for CAD, we confirmed the lipid-lowering and endothelial function regulation effects of BBR and demonstrated the BBR-induced changes in the human gut microbiome and fecal aromatic amino acid pool composition through metagenomics and metabolomics study.

Despite the widespread use of medical therapy in the last decades, CAD remains the leading cause of mortality worldwide. The inflammatory response triggered by vascular injury after percutaneous coronary intervention (PCI) is a central mechanism underlying major adverse cardiac events, among which endothelial dysfunction plays a critical role. Our results showed that BBR reduced circulating inflammatory markers and improved endothelium-dependent flow-mediated dilation in SCAD patients post-PCI, extending prior preclinical evidence of BBR’s vascular benefits (23).

Berberine is an isoquinoline alkaloid, and due to its extremely low bioavailability, it can easily interact with the gut microbiota, which has been implicated as a novel “endocrine organ” that plays an important role in CVDs (24, 25). In this study, we identified a group of acetate- and butyrate-producing bacterial strains that were selectively promoted by BBR treatment. These positive responders are likely the key players for maintaining the mutualistic relationship between the gut microbiota and the human host; promoting this active group of SCFA producers not only enhanced a beneficial function but also maintained a gut environment that keeps detrimental bacteria at bay. The triple association between the gut microbiome, SCFAs, and clinical outcomes suggested a potential microbe-related mechanism underlying the metabolic benefits of BBR.

Through integrative analysis of metagenomics and fecal metabolomics, we identified the pathways involved in aromatic amino acid metabolism as being particularly prominent under BBR treatment. In a large-scale clinical study (*N* > 4,000), aromatic amino acid nutrient precursors (Phe, Tyr, and Trp) had lower circulating levels among individuals with MACE vs. those without; however, phenylacetyl glutamine (PAGln from phenylalanine) and 4-OH-phenyllactic acid (from tyrosine) were independently associated with incident MACE (26). Interestingly, in our study, BBR treatment did not significantly increase the abundance of precursors, whereas the levels of L-DOPA and phenylpropionic acid were markedly elevated. L-DOPA exhibits a complex relationship with CAD, functioning potentially as a risk factor and as a therapeutic agent depending on the context and dosage (27). Moreover, BBR treatment elevated fecal phenylpropionic acid levels, which significantly strengthened the intestinal epithelial barrier via the activation of aryl hydrocarbon receptor (AhR) signaling, which led to the attenuation of intestinal inflammation (28). Although circulating L-DOPA is largely gut–brain–cardiac neuromodulatory, low-dose dopaminergic signaling has been implicated in endothelial NO bioavailability—whether the observed fecal enrichment reflects a local gut–vascular axis rather than systemic exposure warrants dedicated tracer studies. Overall, BBR reshaped the gut microbiota structure, leading to improved aromatic amino acid metabolism and subsequent perturbation of lipid and inflammatory balance.

Our study has several limitations. First, the sample size of this pilot parallel-controlled trial was determined based on the feasibility and detection of exploratory microbiome effects, aligning with comparable BBR human studies (29). Future definitive RCTs will require substantially larger sample sizes. Second, the two groups differ substantially in age and sex distribution; residual confounding by factors, such as smoking, alcohol consumption, dietary habits, and physical activity, cannot be fully excluded, despite extensive clinical annotation. Additionally, missing data for certain clinical parameters precluded between-group comparisons of changes for some outcomes, limiting our analysis to within-group pre-post assessments. Third, the limited cohort size restricted our ability to identify downstream metabolite alterations. Specifically, PAGln—a thrombotic risk marker subject to hepatic glutamine transferase conversion—showed no significant changes. Additionally, despite BBR’s low bioavailability, direct effects on host tissues remain possible.

Using a parallel-controlled study design, we controlled for potential confounding bias from other CAD secondary prevention therapies on gut microbiota. Our findings suggest that CAD may result from the loss or deficiency of beneficial functions within the gut ecosystem—for example, SCFA production. Targeted promotion of these beneficial metabolites through BBR administration may offer a novel ecological strategy for manipulating the gut microbiota to prevent or manage cardiometabolic diseases.

## Data Availability

The data analyzed in this study are available in the Genome Sequence Archive (https://ngdc.cncb.ac.cn/gsa/browse/CRA049888) under accession number CRA049888, associated with BioProject accession number PRJCA027349.
